# Biochemical and Molecular Responses Underlying the Contrasting Phosphorus Use Efficiency in Ryegrass Cultivars

**DOI:** 10.3390/plants12061224

**Published:** 2023-03-08

**Authors:** Sofía Pontigo, Leyla Parra-Almuna, Ana Luengo-Escobar, Patricia Poblete-Grant, Adriano Nunes-Nesi, María de la Luz Mora, Paula Cartes

**Affiliations:** 1 Center of Plant Soil Interaction and Natural Resources Biotechnology, Scientific and Technological Bioresource Nucleus (BIOREN-UFRO), Universidad de La Frontera, Avenida Francisco Salazar 01145, P.O. Box 54-D, Temuco 4780000, Chile; 2 Departamento de Ciencias Químicas y Recursos Naturales, Facultad de Ingeniería y Ciencias, Universidad de La Frontera, Avenida Francisco Salazar 01145, P.O. Box 54-D, Temuco 4780000, Chile; 3 Instituto de Investigaciones Agropecuarias, INIA Carillanca, km 10 camino Cajón-Vilcún s/n, Temuco P.O. Box 929, Chile; 4 Departamento de Biologia Vegetal, Universidade Federal de Viçosa, Viçosa 36570-900, MG, Brazil

**Keywords:** phosphorus acquisition efficiency, phosphorus utilization efficiency, phosphate transporters, acid phosphatases, ryegrass

## Abstract

Improving plant ability to acquire and efficiently utilize phosphorus (P) is a promising approach for developing sustainable pasture production. This study aimed to identify ryegrass cultivars with contrasting P use efficiency, and to assess their associated biochemical and molecular responses. Nine ryegrass cultivars were hydroponically grown under optimal (0.1 mM) or P-deficient (0.01 mM) conditions, and P uptake, dry biomass, phosphorus acquisition efficiency (PAE) and phosphorus utilization efficiency (PUE) were evaluated. Accordingly, two cultivars with high PAE but low PUE (Ansa and Stellar), and two cultivars with low PAE and high PUE (24Seven and Extreme) were selected to analyze the activity and gene expression of acid phosphatases (APases), as well as the transcript levels of P transporters. Our results showed that ryegrass cultivars with high PAE were mainly influenced by root-related responses, including the expression of genes codifying for the P transporter *LpPHT1;4*, purple acid phosphatase *LpPAP1* and APase activity. Moreover, the traits that contributed greatly to enhanced PUE were the expression of *LpPHT1;1/4* and *LpPHO1;2*, and the APase activity in shoots. These outcomes could be useful to evaluate and develop cultivars with high P-use efficiency, thus contributing to improve the management of P in grassland systems.

## 1. Introduction

Phosphorus (P) is an essential macronutrient for plants, but it is commonly deficient in most agricultural soils because of its low availability and reduced efficiency of P fertilizers [1,2]. Phosphorus occurs in two chemical forms in soil, i.e., organic (e.g., phytate and phosphoesters) and inorganic (orthophosphate; Pi) forms; only the latter can be assimilated by plants. Soil P can be strongly affected by chemical factors, such as the formation of insoluble complexes with cations including iron (Fe), aluminum (Al) or calcium (Ca), as well as by sorption of P on mineral surfaces and organic matter in a pH-dependent manner [3]. To counteract such limitations, an excessive application of P-containing fertilizers to soils is usually performed, but only 20–30% of the P supplied is taken up by plants. In addition, this practice can contribute to the enrichment of water bodies with nutrients that cause eutrophication, leading to environmental constraints [4]. An even greater concern is the depletion of the global P reserves used to produce P fertilizers, whose scarcity can trigger several problems for agricultural production and food security [5]. Consequently, the cost of P fertilizer has been steadily rising over the last decades due to the demand driven by the limitation of P resources and the increasing human population [6,7]. In this context, new adaptive strategies that lead to the adequate and sustainable management of P in agriculture are currently needed. 

An improvement of plant P efficiency is a promising approach for developing sustainable agriculture practices. Plant P-use efficiency includes both the P-acquisition efficiency (PAE) and the internal utilization efficiency (PUE). Phosphorus acquisition efficiency is the ability of plants to take up P from the soil and is commonly expressed as the relative difference of P acquired under low P versus high P availability [8]. As reviewed by Han et al. (2022) [9], PAE has been mainly associated with: (1) the modification of root system architecture; (2) the induction of root exudation of organic acid anions, protons, and P-releasing enzymes; (3) mycorrhizal associations; and (4) the upregulation of P-specific root transporters. On the other hand, the capacity to produce a large amount of biomass per unit of P accumulated is known as PUE, which is conferred by an efficient internal use of P. Phosphorus utilization efficiency can be achieved by an effective translocation and remobilization of P as well as by an improvement of the processes related to P releasing from inorganic and organic pools within the plant. Although the relevance of PAE and PUE are still under discussion, scientific evidence has suggested that plants with high PUE, rather than high PAE, may be more promising for enhancing P efficiency in intensive cropping systems [8]. Consequently, the improvement of plant traits related to PUE may result in a more effective use of P resources, considering that plants will acquire less P, thus reducing both P fertilizer requirements and P removal from soils [10,11]. In this way, an important aspect to enhance PUE is the effective plant scavenging and recycling of P mediated by intracellular acid phosphatases (APases), specially by purple acid phosphatases (PAPs), which catalyze the hydrolysis of a wide range of phosphate esters and anhydrides [12,13,14]. Although it has been documented that most PAPs can be induced by P deficiency, little is known about the implication of PAPs on plant P use efficiency. 

Phosphate transporters (PHTs) also play an important role in P mobilization within the plant [15]. Phosphate transporters are classified into five major families in plants (PHT1, PHT2, PHT3, PHT4, and PHT5), differentiated by their sub-cellular localization and functional properties [16]. Phosphate transporters belonging to the PHT1 subfamily are the most relevant for P uptake by roots and its remobilization inside the plant, whereas members of the phosphate efflux transporter (PHO1) mediate the loading of Pi into the xylem and its distribution within the plant [17]. An efficient plant P transport system seems to be essential to mediate a higher PUE, and some studies suggest that a higher expression of P transporters might lead to a greater PUE in different plant species including barley [18], soybean [19] and rice [20]. In this way, both the genetic variation in gene expression or activity of P transporters and their possible relationship with either PAE or PUE deserve to be investigated. 

Perennial ryegrass (*Lolium perenne* L.), one of the main pasture species in temperate areas of the world, and the dominant forage specie for beef and dairy production in Southern Chile, is also highly dependent of P fertilization [21]. Accordingly, increasing P fertilizer use efficiency is also a desirable trait for grassland systems. Nevertheless, relatively few studies have focused on investigating the biochemical and molecular responses of ryegrass to P deficiency [22,23,24], and even less to associating these aspects with P use efficiency. Thus, this study was conducted to identify ryegrass cultivars with contrasting P use efficiency, and to assess their biochemical and molecular responses in terms of the activity and gene expression of APases, as well as the transcript level of genes involved in the uptake, translocation, and remobilization of P.

## 2. Results

### 2.1. Phosphorus Concentration and Uptake

Shoot P concentration ranged between 3.07 ± 0.01 g kg^−1^ DW and 6.03 ± 0.37 g kg^−1^ DW in cultivars grown at optimal P supply (0.1 mM P). However, P concentration decreased in shoots and roots of all ryegrass cultivars when P dose was reduced to 0.01 mM (Table 1). Under low P conditions, shoot P concentration was significantly lower in 24 Seven and Extreme (0.77 ± 0.03 g kg^−1^ DW and 0.84 ± 0.02 g kg^−1^ DW, respectively), whereas Ansa and Stellar displayed the highest shoot P concentrations (1.79 ± 0.08 g kg^−1^ DW and 1.99 ± 0.07 g kg^−1^ DW, respectively). A similar trend was observed in roots of P-deficient plants, which showed a P concentration ranged from 0.87 ± 0.02 g kg^−1^ DW (24Seven) to 2.07 ± 0.09 g kg^−1^ DW (Stellar). Regarding the plant P content, all ryegrass cultivars preferentially accumulated P in the shoots irrespective of added P. Accordingly, Nui showed the highest shoot P content (53.0 ± 3.15 g pot^−1^) under optimal P supply, whereas the minor P accumulation was observed in Ansa (24.5 ± 1.73 g pot^−1^) at the same P condition. At low P levels, the shoot P content among cultivars fluctuated between 4.52 ± 0.06 g pot^−1^ and 6.78 ± 0.36 g pot^−1^ of 24Seven and Expo, respectively. For roots, Extreme exhibited the highest P content at either adequate (9.87 ± 0.22 g pot^−1^) or deficient (3.20 ± 0.19 g pot^−1^) P supply. In contrast, a lower root P content was found in Ansa grown at both 0.01 mM P (1.55 ± 0.05 g pot^−1^) and 0.1 mM P (3.12 ± 0.35 g pot^−1^). 

### 2.2. Growth Responses of Ryegrass Plants to P Treatments 

**Figure 1:**
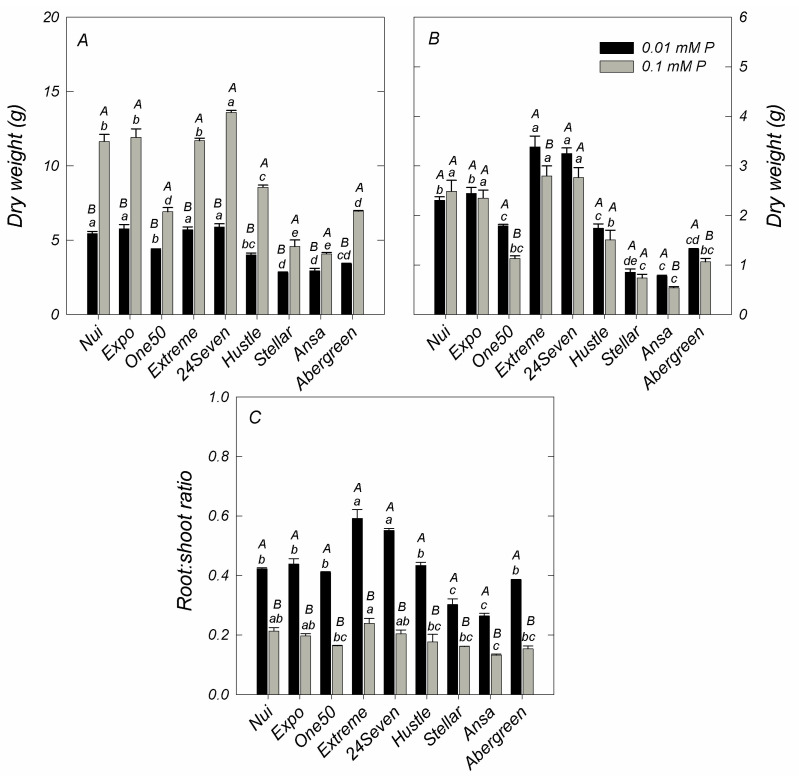
Dry weight in shoots (**A**) and roots (**B**), and root:shoot ratio (**C**) of different ryegrass cultivars hydroponically grown at optimal (0.1 mM P) and deficient (0.01 mM P) P conditions. Values represent the mean (n = 3) ± standard error. Different uppercase letters indicate statistically significant differences (Tukey’s HSD at *p* ≤ 0.05) between P treatments for the same cultivar. Different lowercase letters indicate statistically significant differences (Tukey’s HSD at *p* ≤ 0.05) among ryegrass cultivars at the same P treatment.

As shown in Figure 1, the ryegrass cultivars showed significant variations in dry weight (DW) of shoots at either sufficient or deficient P levels. In general, shoot DW of cultivars supplied with 0.1 mM P varied from 4.08 ± 0.09 g pot^−1^ (Ansa) to 13.60 ± 0.13 g pot^−1^ (24Seven), with an average of 8.8 ± 1.15 g pot^−1^ for all examined cultivars. Shoot DW significantly decreased when a deficient P supply was imposed in all ryegrass cultivars (Figure 1A). In fact, a direct correlation between shoot DW and shoot P accumulation (r = 0.795, *p* ≤ 0.01) was found. Although 24Seven exhibited the highest reduction of shoot biomass at low P, it produced the major shoot DW at either 0.1 or 0.01 mM P. In contrast, Stellar and Ansa cultivars produced less shoot biomass than other cultivars grown at both P conditions. Nevertheless, these cultivars showed the lower decrease of shoot dry matter under low P respect to the control (Figure 1A). Different to shoot, low P levels triggered an increment of root DW in most cultivars, which reached an average root DW of 1.98 ± 0.31 g pot^−1^ (Figure 1B). Accordingly, 24Seven and Extreme showed the highest root biomass production under P deficiency, whereas the lowest root DW was observed in Stellar and Ansa at 0.01 mM P. In this way, all ryegrass cultivars grown at low P supply increased the root: shoot ratio at least 1.9-fold compared to those supplied with an optimal P dose (Figure 1C). Moreover, a positive correlation between root: shoot ratio and root DW (r = 0.583, *p* ≤ 0.01) was found. At deficient P condition, 24Seven and Extreme displayed the maximum root: shoot ratio, whereas the lowest root: shoot ratio was observed in Ansa and Stellar. 

### 2.3. Phosphorus Acquisition and Utilization Efficiency 

**Figure 2:**
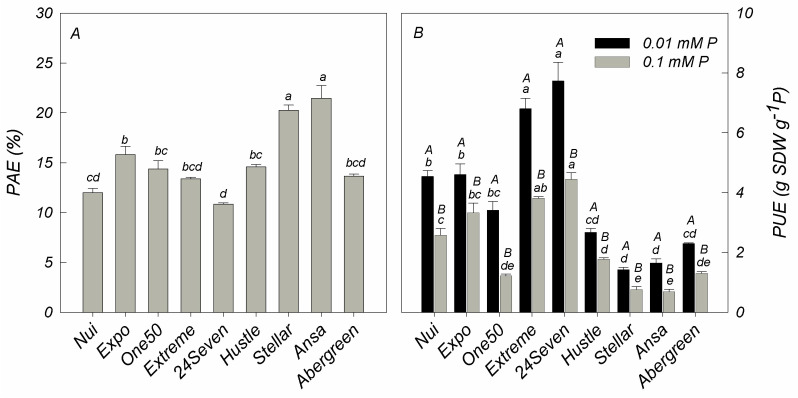
Phosphorus acquisition efficiency (PAE; (**A**)) and phosphorus utilization efficiency (PUE; (**B**)) in different ryegrass cultivars hydroponically grown at optimal (0.1 mM P) and deficient (0.01 mM P) P conditions. Values represent the mean (n = 3) ± standard error. Different uppercase letters indicate statistically significant differences (Tukey’s HSD at *p* ≤ 0.05) between P treatments for the same cultivar. Different lowercase letters indicate statistically significant differences (Tukey’s HSD at *p* ≤ 0.05) among ryegrass cultivars at the same P treatment.

As shown in Figure 2, the ratio of shoot P content in deficient and sufficient P conditions of all cultivars was calculated for PAE, which ranged from 11% for 24Seven to 21% for Ansa. Among all cultivars, Ansa (21%) and Stellar (20%) showed the major PAE values (Figure 2A). In addition, PUE, expressed as shoot DW produced per unit of P concentration in aboveground plant, decreased with increasing P application in all ryegrass cultivars (Figure 2B). Unlike PAE, the highest PUE was observed in 24Seven and Extreme, whereas the lowest PUE was detected in Ansa and Stellar irrespective of P condition (Figure 2B). Accordingly, a negative correlation was found between PAE and PUE at low P level (r = −0.726, *p* ≤ 0.01) and optimal P supply (r = −0.692, *p* ≤ 0.01). Based on these results, two cultivars with high PUE and low PAE (24Seven and Extreme) and two cultivars with low PUE and high PAE (Ansa and Stellar) were selected in order to evaluate biochemical and molecular responses likely involved in their contrasting P efficiency.

### 2.4. Gene Expression Analysis of P Transporters 

**Figure 3:**
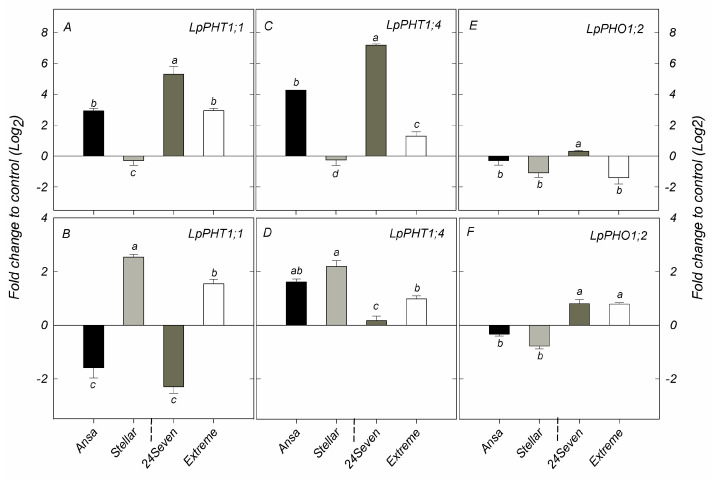
Expression analysis of phosphate transporters *LpPHT1;1* (**A**,**B**); *LpPHT1;4* (**C**,**D**); and *LpPHO1;2* (**E**,**F**) genes determined by qRT-PCR in shoots and roots of ryegrass cultivars hydroponically grown at optimal (0.1 mM P) and deficient (0.01 mM P) P conditions. All data were normalized to geometric mean value from *LpeEF1α(h)* and *LpeEF1α(s)* housekeeping genes and expressed as fold change of control treatment (0.1 mM P). Log2 was performed to better understand the regulation of these genes. Values represent the mean (n = 3) ± standard error. Different letters indicate statistically significant differences (Tukey’s HSD at *p* ≤ 0.05) between cultivars.

**Figure 4:**
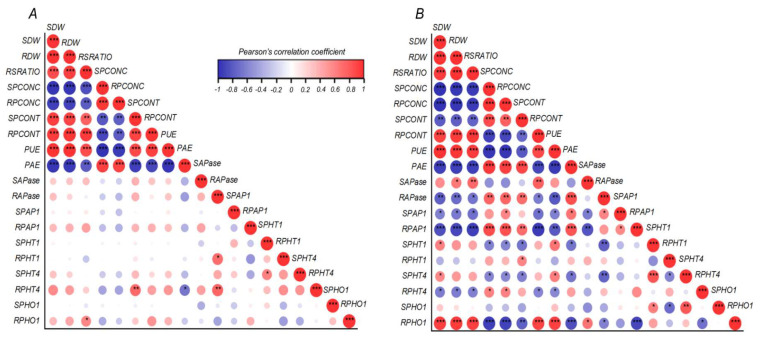
Pearson’s correlation coefficient matrix among growth, biochemical and molecular parameters of ryegrass cultivars hydroponically grown at optimal (0.1 mM P; (**A**)) and deficient (0.01 mM P; (**B**)) P conditions. Pearson’s coefficient * *p* ≤ 0.05, ** *p* ≤ 0.01, *** *p* ≤ 0.001. Positive and negative correlations are distinguished by blue and red colors, respectively. Abbreviations: phosphorus acquisition efficiency (PAE), phosphorus utilization efficiency (PUE), shoot dry weight (SDW), root dry weight (RDW), root:shoot ratio (RSRATIO), shoot P concentration (SPCONC), root P concentration (RPCONC), shoot P content (SPCONT), root P content (SPCONT), shoot *LpPHT1;1* expression (SPHT1), root *LpPHT1;1* expression (RPHT1), shoot *LpPHT1;4* expression (SPHT4), root *LpPHT1;4* expression (RPHT4), shoot *LpPHO1;2* expression (SPHO), root *LpPHO1;2* expression (RPHO), shoot *LpPAP1* expression (SPAP1), root *LpPAP1* expression (RPAP1), shoot APase activity (SAPase) and root APase activity (RAPase).

The relative expression of genes involved in the uptake and transport of P, such as *LpPHT1;1*, *LpPHT4;1* and *LpPHO1;2*, were evaluated in shoots and roots of the four ryegrass cultivars selected above (Figure 3). The transcript levels of *LpPHT1;1* and *LpPHT1;4* were enhanced in shoots of ryegrass cultivars grown at low P supply, with the exception of Stellar that displayed no changes in the expression of these genes between P rates (Figure 3A,C). Accordingly, 24Seven showed the highest transcript levels of *LpPHT1;1* and *LpPHT1;4* in shoots respect to other cultivars. Interestingly, the gene expression of both *LpPHT1;1* and *LpPHT4;1* in shoots was positively correlated with PUE (r = 0.668, *p* ≤ 0.01 and r = 0.628, *p* ≤ 0.01, respectively) at low P conditions Figure 4B). Nevertheless, a negative relationship between the expression of these genes in shoots and PAE was also observed (r = −0.666, *p* ≤ 0.01 and r = −0.671, *p* ≤ 0.01, respectively). In addition, the relative expression of *LpPHO1;2* was decreased in shoots of cultivars Extreme and Stellar grown under P deficiency, whereas no significant differences in the transcript level of this gene were found in 24Seven and Ansa (Figure 3E). At low P conditions, a down-regulation of *LpPHT1;1* was observed in roots of cultivars 24Seven and Ansa. In contrast, the expression of *LpPHT1;1* was induced in roots of Extreme and Stellar grown at 0.01 mM P (Figure 3B). Similarly, *LpPHT1;4* was upregulated by low P supply in roots of all ryegrass cultivars, with the highest expression level observed in Ansa and Stellar (Figure 3D). Conversely, the expression of *LpPHO1;2* was downregulated by P deficiency in roots of Ansa and Stellar, whereas the transcript levels of this gene enhanced in roots of 24Seven and Extreme cultivated at low P dose (Figure 3F). For roots, this outcome coincided with the strong positive correlation found between the expression of *LpPHO1;2* and PUE (r = 0.921, *p* ≤ 0.01), whereas the expression of this gene and PAE were negatively related (r = −0.848, *p* ≤ 0.01) at low P (Figure 4B).

### 2.5. Phosphatase Activity and Gene Expression of PAP1 

**Figure 5:**
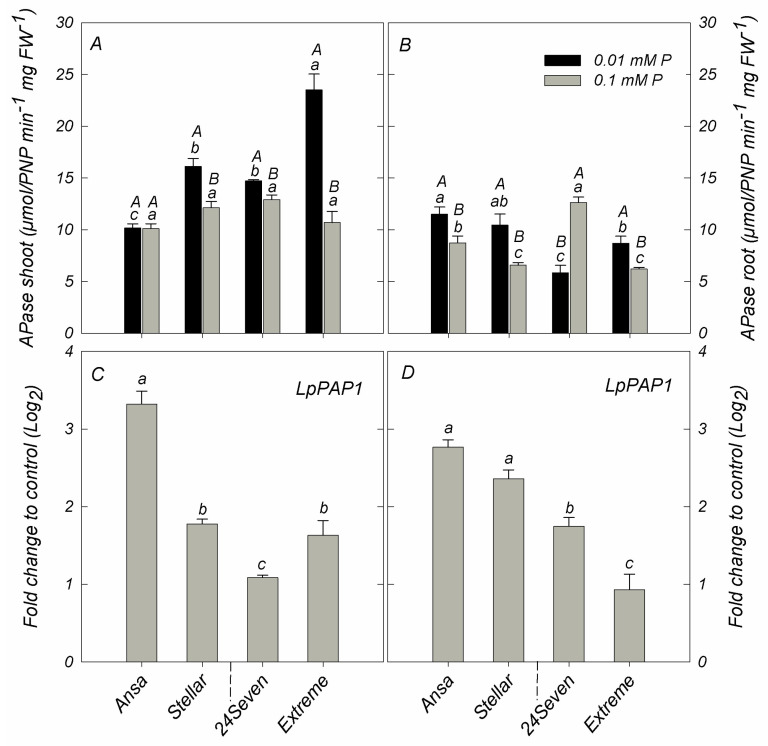
Acid phosphatase activity in shoots (**A**); and roots (**B**); and expression analysis of purple acid phosphatase *LpPAP1* in shoots (**C**); and roots (**D**) of ryegrass cultivars hydroponically grown at optimal (0.1 mM P) and deficient (0.01 mM P) P conditions. All data were normalized to geometric mean value from *LpeEF1α(h)* and *LpeEF1α(s)* housekeeping genes and expressed as fold change of control treatment (0.1 mM P). Log2 was performed to better understand the regulation of these genes. Values represent the mean (n = 3) ± standard error. Different uppercase letters indicate statistically significant differences (Tukey’s HSD at *p* ≤ 0.05) between P treatments for the same cultivar. Different lowercase letters indicate statistically significant differences (Tukey’s HSD at *p* ≤ 0.05) among ryegrass cultivars at the same P treatment.

Apparent differences in APase activity were found among ryegrass cultivars grown at either 0.01 and 0.1 mM P (Figure 5A,B). Excluding Ansa and 24Seven, shoot APase activity was increased in cultivars grown under low P condition. Consequently, Extreme showed the higher shoot APase activity respect to other cultivars, increasing twice under P deficiency respect to sufficient P supply (Figure 5A). Compared to the control, low P supply also increased the root APase activity in all ryegrass cultivars with the exception of 24Seven, which showed a decrease of root APase activity by about 46.3% (Figure 5B). In this regard, root APase activity was positively correlated with PAE (r = 0.846, *p* ≤ 0.01), but negatively related with PUE (r = −0.807, *p* ≤ 0.01) (Figure 4B). Additionally, the reduction in P supply increased the expression level of *LpPAP1* in shoots with a more noticeable effect in cultivar Ansa than other cultivars (Figure 5C). A similar expression pattern was observed for *LpPAP1* in roots, which increased by around 2.8-fold in Ansa, 2.4-fold in Stellar, 1.8-fold in 24Seven and 1.0-fold in Extreme (Figure 5D). Accordingly, a positive correlation between the expression level of *LpPAP1* and PAE at low P was detected in both shoots (r = 0.661, *p* ≤ 0.01) and roots (r = 0.831, *p* ≤ 0.01). In contrast, *LpPAP1* gene expression was negatively related with PUE in shoots (r = −0.608, *p* ≤ 0.01) and roots (r = −0.812, *p* ≤ 0.01) under P-deficient conditions (Figure 4B).

### 2.6. Principal Component Analysis (PCA)

**Figure 6:**
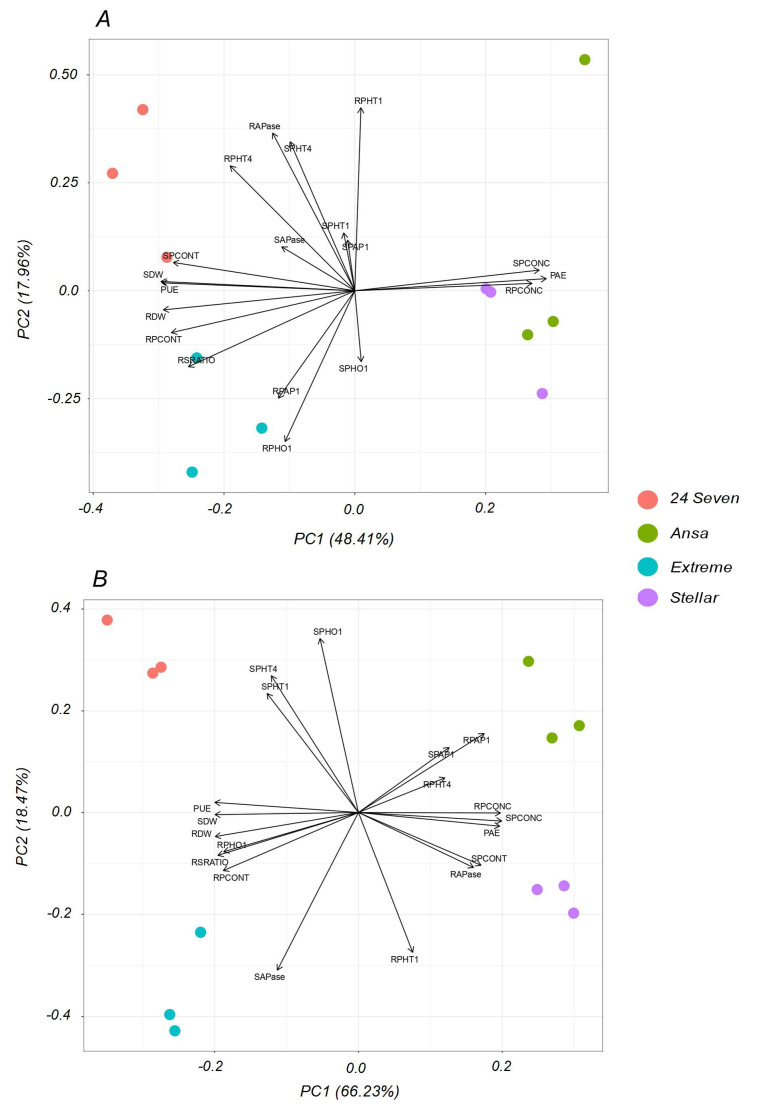
Principal component analysis (PCA) of different traits of four ryegrass cultivars hydroponically grown at optimal (0.1 mM P; (**A**)); and deficient (0.01 mM P; (**B**)) P conditions. Numbers in parentheses show the percent of the total variation explained by the PC1 and PC2, both representing 66.37% for optimal P and 84.7% for P deficiency of the total variance. Biplot vectors are trait factor loadings, whereas the position of individual cultivars is shown in colored circles. Abbreviations: phosphorus acquisition efficiency (PAE), phosphorus utilization efficiency (PUE), shoot dry weight (SDW), root dry weight (RDW), root:shoot ratio (RSRATIO), shoot P concentration (SPCONC), root P concentration (RPCONC), shoot P content (SPCONT), root P content (SPCONT), shoot *LpPHT1;1* expression (SPHT1), root *LpPHT1;1* expression (RPHT1), shoot *LpPHT1;4* expression (SPHT4), root *LpPHT1;4* expression (RPHT4), shoot *LpPHO1;2* expression (SPHO), root *LpPHO1;2* expression (RPHO), shoot *LpPAP1* expression (SPAP1), root *LpPAP1* expression (RPAP1), shoot APase activity (SAPase) and root APase activity (RAPase).

To explore for possible common factors that would explain the observed correlations, a PCA based on all measured traits was performed individually per each P treatment (Figure 6). Under P optimal conditions (Figure 6A), the first two components explained 66.37% of the total variance, which was enough to explain the data variability at sufficient P supply. According to the individual representation of the cultivars, they were separated in two group by the first axis being Ansa-Stellar close-by and followed a positive direction with PAE, shoot P concentration (SPCONC) and root P concentration (RPCONC). The group of 24Seven-Extreme was positively associated to PUE, shoot P content (SPCONT), root:shoot ratio (RSRATIO), root *LpPHO1;2* expression (RPHO1), root *LpPHT1;4* expression (RPHT4) and root APase activity (RAPase). The quantitatively important variation (66.23% for PC1 and 18.47% for PC2), which was 84.7% of the total variance showed greatly explanation of data variability under low P condition (Figure 6B). The PCA analysis exhibited a clear separation of each cultivar, showing that Stellar was highly associated to root *LpPHT1;1* expression (RPHT1), RAPase and SPCONT, whereas Ansa displayed a greater relation with root *LpPAP1* expression (RPAP1), shoot LpPAP1 expression (SPAP1) and RPHT4. On the other hand, 24Seven was strongly associated to the expression of P transporters in shoots (*LpPHO1;1*, *LpPHT1;1* and *LpPHT1;4*), whereas Extreme was associated principally to APase activity in shoots (SAPase). 

## 3. Discussion

Phosphorus (P) is an essential nutrient required for most of metabolic processes controlling the growth and development of plants [3,25]. In order to maintain P homeostasis, plants have evolved two distinct adaptive strategies to facilitate an efficient P acquisition (PAE) as well as to utilize efficiently internal P (PUE). PAE and PUE are regulated by a complex genetic network that further can differ among plant species and cultivars. We evaluated nine ryegrass cultivars in terms of their P uptake, dry matter production, PAE and PUE at either optimal or P deficient conditions. In general, all ryegrass cultivars reduced the P content and shoot biomass in response to P deficiency (Table 1, Figure 1A,B); nevertheless, differential PAE and PUE were found between them (Figure 2). Among all cultivars, Ansa and Stellar exhibited the highest PAE (Figure 2A), but the lowest PUE due to the reduced biomass production by unit of P acquired at both P growing conditions (Figure 2B). Contrarily, cultivars 24Seven and Extreme displayed the highest PUE values, but the lowest PAE irrespective of P addition levels (Figure 2A,B). Although it has been reported that the contribution of PAE and PUE can be dependent on P availability as well as plant species and genotypes, evidence has indicated that plants with high PUE, rather than high PAE, may be more promising for enhancing P efficiency in intensive cropping systems [8]. In this way, our results suggest that an improved P use efficiency in ryegrass could be more related to PUE than PAE, contributing to maintain higher plant biomass at either optimal or limited P supply. In fact, it has been proposed that genotypes producing more yield, at either adequate or deficient soil P availability, are greatly desirable because they can fit into a wide range of P environments without compromising production [26]. 

In our study, ryegrass cultivars showing the highest PUE also were associated with the greatest root:shoot ratios at low P conditions, which was supported by the strong positive correlation between PUE and root:shoot (r = 0.885, *p* ≤ 0.01). In agreement, Huang et al. (2011) [18] found similar results in barley genotypes, denoting that a high carbohydrate partitioning into roots occurs simultaneously with a high PUE. Despite PAE and PUE are independent traits that could be improved simultaneously [27,28], we found negative correlation between PAE and PUE (r = −0.714, *p* ≤ 0.01) in agreement with previous findings [18,29]. In this line, PUE is generally lower in genotypes with high PAE as a result of higher shoot P concentrations [30]. Accordingly, plants with high P concentrations could store large amounts of P in vacuoles, consequently producing a negative effect on PUE [31]. Moreover, a quantitative trait locus with the potential to influence PUE have mainly been found in plants with low PAE, indicating that increased P acquisition does not necessarily lead to a more internal P utilization efficient genotype [32]. 

The selection and identification of cultivars with an improved acquisition and remobilization of P is a promising approach to cope with the negative impact of P deficiency on plants. In this context, we further analyzed some biochemical and molecular traits that could be involved in the contrasting P use efficiency in ryegrass cultivars. Such variations might be related to differential gene expression of P transporters, including *LpPHT1;1*, *LpPHT1;4* and *LpPHO1;2*. Previous functional characterization of two PHT1 from ryegrass revealed that *LpPHT1;1/4* play a key role in P uptake and P remobilization under both sufficient and deficient P conditions [33]. In general, under P limitation, an upregulation of *LpPHT1;1* and *LpPHT1;4* was observed in most of ryegrass cultivars (Figure 3). The only exception was *LpPHT1;1*, which did not display a clear trend in the expression pattern in the roots. These findings were consistent with a previous report showing that the expression of *LpPHT1;4*, but not *LpPHT1;1*, was strongly influenced by P deficiency denoting different regulatory pathways [33]. Interestingly, we also found the highest gene expression of *LpPHT1;1 and LpPHT1;4* in shoots of 24Seven, which was the cultivar that displayed the major PUE values (Figure 3A,C). Thus, an efficient P mobilization through the shoots mediated by P transporters could be the major determinant of high PUE for this cultivar. Moreover, this finding coincided with the positive correlation between the gene expression of these P transporters in shoots and PUE observed here (Figure 4B). The relationship between P transporters and PUE has not been fully understood yet. In agreement with our results, an enhanced PUE has been associated to a high expression of *HvPHT1;6* and *HvPHT1;3* in barley genotypes [18]. Furthermore, Song et al. (2014) [19] found that overexpressing *PHT1;1* from soybean in tobacco lead to an increment in PUE at optimal or P deficient supply. On the other hand, PHO1, an essential P transporter mainly implicated in P transport from root to shoot by loading P into the xylem has also been related to an improved PUE [16,34]. Indeed, *PHO1;2*, one member of the PHO1 family, improved PUE under low P availability when was overexpressed in rice [35]. Similarly, we found an increment in the expression level of *LpPHO1;2* in roots of the cultivars showing the highest PUE values, whereas a down-regulation of this gene was observed in shoots and roots of the cultivars exhibiting the lowest PUE (Figure 3E,F). Changes in the expression level of *PHO1;2* has also been reported between barley cultivars with contrasting P efficiency showing a predominant upregulation of *PHO1;2* in the P-efficient cultivar under poor P conditions [36]. 

Another important strategy of plants for enhancing PAE or PUE to maintain cellular P homeostasis is the induction of both intra- and extracellular activity of APases [37]. Among them, intracellular PAPs are the major class of plant APases that play an essential role remobilizing Pi from cellular reserves, whereas secreted ones hydrolyze Pi from P organic compounds in apoplast and rhizosphere [13,14]. Accordingly, we found an induction of shoot APase activity only in Extreme and Stellar grown at low P, indicating that APases activity was not related to P status in shoots (Figure 5A). However, an increment of APase activity in the roots of almost all ryegrass cultivars was consistent with the positive correlation between root APase activity and either root P accumulation or PAE (Figure 5B). Interestingly, 24Seven was the only ryegrass cultivar that decreased the APase activity in roots subjected to P limitation. Although APase activity is often increased as an adaptive response to low P availability [14,38], genotypic variations in APase activity observed in this study could denote the existence of different strategies for improving the acquisition or remobilization of P under low P stress in ryegrass. These variations could be explained by considering that a wide range of plants, genotypes and even tissues can show non-specific APase activity, with substantial differences in protein size, tissue or subcellular localization, and in the molecular regulation [12]. An upregulation of *LpPAP1* in both shoots and roots was also observed in all ryegrass cultivars subjected to P deficiency (Figure 5C,D), indicating that the expression of this gene is highly modulated by P status in ryegrass. Similar results showing an increment of levels of PAPs transcripts under P limitation have been reported in other plant species, including Arabidopsis [39], tomato [40], rice [41] and maize [42]. The higher expression of *LpPAP1* in roots of Ansa and Stellar also coincided with the lower root P accumulation in these cultivars respect to 24Seven and Extreme. Moreover, both the APase activity and the expression of *LpPAP1* in roots of Ansa and Stellar were positively correlated with PAE suggesting that in these cultivars, APases associated with root surface may play a pivotal role in P acquisition efficiency.

Biochemical and molecular differences among ryegrass cultivars found in this study were also supported by PCA analysis (Figure 6), which confirmed the genetic variability between the selected cultivars in terms of PAE and PUE during P stress. In general, the cultivars with high PAE (Ansa and Stellar) were influenced by root-related responses, whereas cultivars with high PUE (24Seven and Extreme) were closely associated to attributes derived from shoots. Interestingly, our results showed a clear separation among ryegrass cultivars under limited P condition. Accordingly, improved PAE in Ansa was mostly related to the expression of genes codifying for *LpPHT1;4* in roots and *LpPAP1* in shoots and roots. In addition, the root APase activity contributed greatly to enhanced PAE in Stellar. On the other hand, the main traits that influenced the high PUE in 24Seven were the expression of P transporters *LpPHT1;1/4* and *LpPHO1;2* in shoots, whereas those in Extreme contributed significantly to shoot APase activity. 

## 4. Conclusions

Taken together, our study confirms genetic variations in P use efficiency among different ryegrass cultivars grown under P deficiency. Indeed, the PAE and PUE ability of each selected cultivar was clearly demonstrated through the differential expression of P-related genes. Consequently, our findings contribute to identify P efficient cultivars that could require less P to achieve the expected yield under a P stress scenario. This study also provides important evidence about the role of P transporters and APases on P use efficiency, an aspect little explored in forage species such as ryegrass which are highly dependent on P nutrition. Our findings could be also useful in evaluating and developing cultivars with high P use efficiency leading to improvements in the management of P in grassland systems. Thus, unravelling the biochemical or molecular nature of the relationship between P transporters/APases and PAE/PUE might provide a valuable insight for future plant-breeding efforts.

## 5. Materials and Methods

### 5.1. Plant Material and Growth Conditions

Nine perennial ryegrass (*Lolium perenne* L.) cultivars (Nui, Expo, One50, 24Seven, Extreme, Stellar, Ansa, Hustle and Abergreen) of New Zealand origin and currently in the Chilean pasture seed market, were evaluated in this study. The seeds of all ryegrass cultivars, were rinsed with 2% *v*/*v* sodium hypochlorite for 15 min, washed several times with distilled water and then germinated on moist filter paper in a growth chamber at 21 °C. After 10 days of germination, the seedlings were transferred to continuously aerated hydroponic culture pots (7 L) using a basal nutrient solution proposed by Taylor and Foy (1985) [43]. After an acclimation period of 7 days, two P levels were applied (0.1 mM, P-optimal; and 0.01 mM P, P-deficiency; supplied as K_2_HPO_4_) over 21 days. Potassium (K) was supplied as KCl to maintain equal concentrations of K in solution. Plants were grown in a greenhouse using a completely randomized factorial design with three replicates per treatment under controlled conditions (16 h/8 h [light/darkness] photoperiod at 20 °C and 70–80% relative humidity). During the experiment, the pH of the nutrient solution was adjusted to 6.0 and checked daily. After 21 days of cultivation, root and shoot samples were collected in the middle of light period, snap-frozen with liquid nitrogen and stored at −80 °C for further enzymatic and gene expression analysis. 

### 5.2. Plant Growth and P Determination

Subsamples of fresh material were oven dried at 65 °C for 48 h to determine dry weight (DW) and the concentration of P in plant tissues. Shoots and roots were ashed in a muffle at 500 °C for 10 h and afterward digested with 2 M HCl and filtered to determine P concentrations. The P concentration was measured by the molybdovanadate method, according to Sadzawka et al. (2004) [44]. A molybdovanadate solution was prepared by mixing equal parts 8 mM NH_4_VO_3_, 1.5 mM (NH_4_)_6_Mo_7_O_24_ * 4H_2_O and 1.5 M HNO_3_. Filtered solutions were mixed with 4 mL of the molybdovanadate solution, maintained for 1 h at room temperature and then spectrophotometrically measured at 466 nm. Two reference samples with known P concentrations were included for each analytical run as internal controls. 

Using plant P concentration and DW data, phosphorus acquisition efficiency (PAE) and phosphorus utilization efficiency (PUE) were calculated as reported by López-Arredondo et al. (2014) [25] and Siddiqi and Glass (1981) [45], respectively.

$$\mathrm{PAE}\;(\%)=\frac{\mathrm{Shoot}\;P\;\mathrm{content}\;\left(\mathrm{deficient}\;P\right)}{\mathrm{Shoot}\;P\;\mathrm{content}\;\left(\mathrm{optimal}\;P\right)}\times 100$$

$$\mathrm{PUE}\;(g\;\mathrm{SDW}\;g^{-1}\;P)=\frac{\mathrm{Shoot}\;\mathrm{DW}\;\left({g\;\mathrm{pot}}^{-1}\right)}{\mathrm{Shoot}\;P\;\mathrm{concentration}\;\left({g\;\mathrm{kg}}^{-1}\right)}$$

### 5.3. Intracellular Acid Phosphatase Activity

For the determination of intracellular acid phosphatase (APase) activity, root and shoot tissues (50–70 mg of frozen plant material) were homogenized with mortar and pestle in 1 mL sodium acetate extraction buffer (50 mM, pH 5.5). After incubation for 20 min on ice, samples were centrifuged two times at 11,000× *g* at 4 °C for 10 min. For the measurement of APase activity, 100 µL of the supernatant was added to 4.4 mL of sodium-acetate buffer and 600 µL pNPP (0.2% in water) and shaken for 30 min at room temperature. To stop the reaction, 900 µL NaOH were added. For each sample, a reference sample was prepared by adding the NaOH before shaking and the supernatant after shaking. The solution was spectrophotometrically analyzed at 410 nm (Multiskan SkyHigh Microplate Spectrophotometer, Thermoscientific) in triplicates. Reference values were subtracted from the sample values and APase activity was calculated as pNP per mg fresh weight and 1 h against a pNP standard curve.

### 5.4. Gene Expression Analysis

To further extend our knowledge about the role of P efficiency on different ryegrass cultivars, the expression pattern of different genes involved in the transport and scavenging of P were evaluated in shoots and roots of plants grown under deficient and sufficient P conditions. Total RNA was extracted using the NucleoSpin^®^ RNA Plant Kit, according to the manufacturer’s instructions (Macherey-Nagel, Düren, Germany). Total RNA was adjusted to a concentration of 1 μg for the synthesis of the first strand of cDNA using the High-Capacity cDNA Reverse Transcription Kit (Invitrogen, Carlsbad, CA, USA). The gene expression level was analyzed by quantitative real-time reverse transcription polymerase chain reaction (qRT-PCR) on a qPCR Step One™ Plus (Applied Biosystems, Foster City, CA, USA) and with the PowerUp™ SYBR™ Green Master Mix Kit (Agilent, Santa Clara, CA, USA). Specific primers to amplify *LpPHT1;1* (GeneBank accession MF966998) and *LpPHT1;4* (GeneBank accession MF966999) genes were obtained from Parra-Almuna et al. (2018) [23], *LpPAP1* gene from Venkatachalam et al. (2009) [46] and *LpPHO1;2* from NCBI database (GeneBank OP856692). Two housekeeping genes, eukaryotic elongation factor 1 alpha *eEF1α(h)* (GeneBank accession GO924753) and *eEF1α(s)* (GeneBank accession GO924801) were used as internal control (Table 2). The qPCR was evaluated in 20 μL of final volume containing 10 μL of Brilliant II SYBR^®^Master Mix (2×), 1 μL of 1:10 diluted cDNA and 2.4 μL of each gene-specific primer (600 nM). Cycling conditions were 95 °C for 10 min, followed by 40 cycles at 95 °C for 30 s, 59 °C for 1 min, and 72 °C for 5 s. All qRT-PCR were determined on three biological replicates with three technical replicates. The normalized values were subjected to a 2^−ΔΔCt^ method [47].

### 5.5. Data Analysis

The data were statistically evaluated by two-way analyses of variance (ANOVA) using the software SigmaPlot v12.0 (Systat Software Inc., San Jose, CA, USA). A Tukey post-hoc test was used to determine the significance of differences among the means at *p* ≤ 0.05. The relationships among variables were examined using Pearson’s correlation analysis. The resulting *p*-values were corrected using the false discovery rate (FDR) script displayed by the Rbio software (www.biometria.ufv.br; accessed on 15 December 2022). Pearson correlation matrix was constructed using the OriginPro 2023 (OriginLab Corp., Northampton, MA, USA). In order to identify the response variables that explained a higher proportion of the total variance, a principal component analysis (PCA) was performed based on Euclidean distances by default settings of “ggfortify” package displayed for R software (R Foundation for Statistical Computing Version 3.6.3, R Development Core Team 2009–2018 RStudio, Inc., Boston, MA, USA).

## Figures and Tables

**Table 1:** Phosphorus (P) concentrations and P content in shoots and roots of different ryegrass cultivars hydroponically grown at optimal (0.1 mM P) and deficient (0.01 mM P) P conditions. Values represent the mean (n = 3) ± standard error. Different uppercase letters indicate statistically significant differences (Tukey’s HSD at *p* ≤ 0.05) between P treatments for the same cultivar. Different lowercase letters indicate statistically significant differences (Tukey’s HSD at *p* ≤ 0.05) among ryegrass cultivars at the same P treatment.

Cultivar/P Treatment	0.01 mM P	0.1 mM P	0.01 mM P	0.1 mM P
	**Shoots**		**Roots**	
	**P Concentration (g kg^−1^ DW)**			
Nui	1.20 ± 0.02 Bd	4.56 ± 0.31 Abc	1.03 ± 0.11 Bb	3.84 ± 0.33 Ac
Expo	1.22 ± 0.08 Bcd	3.62 ± 0.19 Acd	1.08 ± 0.14 Bb	3.26 ± 0.29 Ac
One50	1.29 ± 0.09 Bbcd	5.68 ± 0.20 Aab	1.13 ± 0.05 Bb	4.37 ± 0.27 Aab
Extreme	0.84 ± 0.02 Be	3.07 ± 0.01 Ad	0.96 ± 0.09 Bbc	3.55 ± 0.18 Ac
24Seven	0.77 ± 0.03 Be	3.07 ± 0.13 Ad	0.87 ± 0.02 Bc	3.11 ± 0.03 Ac
Hustle	1.50 ± 0.02 Bb	4.83 ± 0.15 Abc	1.27 ± 0.02 Bb	4.07 ± 0.16 Abc
Stellar	1.99 ± 0.07 Ba	6.09 ± 0.37 Aa	2.07 ± 0.09 Ba	5.75 ± 0.55 Aab
Ansa	1.79 ± 0.08 Ba	6.03 ± 0.52 Aa	2.01 ± 0.10 Ba	5.80 ± 0.68 Aa
Abergreen	1.48 ± 0.01 Bbc	5.34 ± 0.20 Aab	1.33 ± 0.05 Bb	4.51 ± 0.10 Aab
	**P content (mg pot^−1^)**			
Nui	6.55 ± 0.07 Bab	53.0 ± 3.15 Aa	2.36 ± 0.22 Babc	9.48 ± 0.80 Aa
Expo	6.78 ± 0.36 Ba	42.9 ± 1.58 Ab	2.63 ± 0.36 Bab	7.62 ± 0.64 Aab
One50	5.64 ± 0.33 Bbcd	39.2 ± 2.30 Ab	2.02 ± 0.09 Bbc	4.93 ± 0.36 Acd
Extreme	4.80 ± 0.06 Bde	35.9 ± 0.61 Abc	3.20 ± 0.19 Ba	9.87 ± 0.22 Aa
24Seven	4.52 ± 0.06 Be	41.7 ± 1.34 Ab	2.83 ± 0.03 Bab	8.60 ± 0.58 Aab
Hustle	6.02 ± 0.11 Babc	41.3 ± 1.69 Ab	2.22 ± 0.12 Bbc	6.12 ± 0.71 Abc
Stellar	5.61 ± 0.14 Bbcd	27.7 ± 2.28 Acd	1.77 ± 0.13 Bc	4.18 ± 0.34 Acd
Ansa	5.27 ± 0.31 Bcde	24.5 ± 1.73 Ad	1.55 ± 0.05 Bc	3.12 ± 0.35 Ad
Abergreen	5.07 ± 0.08 Bcde	37.1 ± 1.39 Abc	1.75 ± 0.05 Bc	4.79 ± 0.24 Acd

**Table 2:** Primer sequences used for relative expression analysis of phosphate transporter and purple acid phosphatase genes from ryegrass.

Gene Name	Forward Primer (5′–3′)	Reverse Primer (5′–3′)
*LpPHT1;1*	CCT GGG ATT GCT TTC TCA C	TGG TTG CGT CAT CGT CAT AG
*LpPHT1;4*	AAC CAG CGT ACC AGG ACA AC	GAG GAT GAT GCG CCA GAC
*LpPHO1;2*	TGTTCTACCGCTCAACACGG	AGTAGCACGCTGTAAACTCCA
*LpPAP1*	CCTTGGTTGATCGTCCTGAT	TGGCTCATACATCACCCTCA
*LpeEF1α(h)*	ATG TCT GTT GAG CAG CCT TC	GCG GAG TAT ATA AAG GGG TAGC
*LpeEF1α(s)*	CCG TTT TGT CGA GTT TGG T	AGC AAC TGT AAC CGA ACA TAGC

## Data Availability

Not applicable.
